# Parliament and Publications

**Published:** 1910-12-24

**Authors:** 


### Parliament and Publications.

### APPOINTMENT OF MEDICAL OFFICERS OF HEALTH.

A Memorandum has been issued by the Local Government Board containing some general observations on appointments of medical officers of health and inspectors of nuisances. It is stated therein that it is desirable, wherever practicable, that the person holding the office of medical officer of health should not be engaged in private practice. In the case of smaller districts the advantage of obtaining the services of a man who is not engaged in private practice may frequently be secured, either by combination with neighbouring districts for the appointment of a joint medical officer of health or by the combination of other public appointments with that office. In the Board's view the salary offered should be sufficient to attract men with good qualifications and to retain their services.

### THE HEALTH OF THE NAVY.

The Report of the Health of the Navy for the year 1909 reveals the fact that the general health of the Fleet is improving. Compared with the average for the preceding five years, the case, invaliding and death ratios are lower, and the average loss of service for each person has again dropped from 10.8 to 9.76 days. In a total force of 112,700, the total number of cases of disease and injury entered on the sick list was 72,540, which gives a ratio of 643.65 per 1,000, a decrease of 75.34 as compared with the average ratio for the preceding five years. The total number finally invalided was 1,764. The number of deaths was 362 (258 from disease and 104 from injury), giving a ratio of 3.21 per 1,000, a decrease of .54 in comparison with the average ratio for the last five years. During the year under review no case of smallpox was recorded among over 100,000 men, a large proportion of whom are exposed to considerable risk of infection. Of influenza, 2,280 cases were recorded; of scarlet fever, 138; of enteric fever, 150; of pyrexia, 411; of cholera, 2; of dysentery, 55; of pneumonia, 472; of malaria, 611; of tuberculosis, 320; of rheumatic fever, 814; of diseases of the nervous system, 781; of diseases of the eye, 790; of diseases of the circulatory system, 598; of diseases of the respiratory system, 7,762; of diseases of the digestive system, 11,691; of diseases of the lymphatic and glandular system, 450; of diseases of the urinary and generative system, 935; of diseases of the organs of locomotion, 1,531; of diseases of the connective tissue and skin, 8,268. As to venereal diseases, the total number of cases recorded was 13,474—namely, chancroid, 2,364; syphilis primary, 954; syphilis secondary, 3,278; gonorrhœa and its sequelæ, 6,878. There is very little alteration in the amount of this type of disease, but it is to be noted that the system which now obtains of invaliding cooks, etc., who are rendered entirely unfit for the performance of their duties by the presence of venereal disease, if they are unlikely to recover completely within a reasonable period, tends to increase the number of cases invalided. The routine use of the Wasserman reaction, by confirming the diagnosis in some doubtful cases of syphilis, may also tend to swell the numbers slightly. The China station shows the highest case ratio for venereal diseases with 164.16, the irregular list closely following with 163.46, and the Home station coming next with 143.33. Australia, which in 1908 headed the list, and has almost always shown a high ratio, shows 111.25, an interesting fact in view of the legislative action recently taken in New South Wales.—"Statistical Report of the Health of the Navy," 302; price 1s. 2d.

